# Liberal or restricted fluid administration: are we ready for a proposal of a restricted intraoperative approach?

**DOI:** 10.1186/1471-2253-14-62

**Published:** 2014-08-01

**Authors:** Giorgio Della Rocca, Luigi Vetrugno, Gabriella Tripi, Cristian Deana, Federico Barbariol, Livia Pompei

**Affiliations:** 1 Dipartimento di Scienze Mediche Sperimentali e Cliniche, University of Udine, University of Udine, 33100 Udine, Italy

**Keywords:** Fluid management, Liberal versus restricted, Colloid versus crystalloid, Intraoperative fluid

## Abstract

**Background:**

Fluid management in the perioperative period has been extensively studied but, despite that, “the right amount” still remains uncertain. The purpose of this paper is to summarize the state of the art of intraoperative fluid approach today.

**Discussion:**

In the current medical literature there are only heterogeneous viewpoints that gives the idea of how confusing the situation is. The approach to the intraoperative fluid management is complex and it should be based on human physiology and the current evidence.

**Summary:**

An intraoperative restrictive fluid approach in major surgery may be beneficial while Goal-directed Therapy should be superior to the liberal fluid strategy. Finally, we propose a rational approach currently used at our institution.

## Background

Fluid management in the perioperative period has been extensively studied but, despite that, “the right amount” still remains uncertain. The only scientific evidence that has recently arisen is that a fluid overload (without proper guidance) seems to be a wrong strategy. Once again “devil stay in the details” and a careful differentiation between “fluid maintenance” and “fluid bolus” seems to be necessary to understand. Fluid maintenance should represent direct measurements of baseline evaporation rate from skin, airways and eventually the surgical field. In the last 50 years many studies indicated that fluid loss because of the perspiration in major abdominal surgery was high: (bodyweight + 40) (kg) × 1(ml/kg/h) [1]. Over the years this excessive fluids overutilization has been disguised and rationalized by the effect of anesthetic drugs induced hypotension, given also the reluctance of some clinicians to the use of vasopressor agents. Lamke et al. experimentally evaluated insensible perspiration and showed that it was highly overestimated [2]. The authors calculated that baseline evaporation was approximately 0.5 ml/Kg/h in the awake adult and that it could increase to 1 mL/Kg/h at the most, during large abdominal surgery (including all bowel surgery). They also assessed that the impact of preoperative fasting on the state of the preoperative volemia was negligible [2]. Over the last few years, this circumstances gave rise to two “styles” of anesthesia management: the “liberal” and “restricted” fluid administration strategy depending on the knowledge and beliefs of that single anesthetist. But, what does “liberal approach” means compared to a “restricted one”? A standardized quantitative definition of these still remain uncertain; in the current medical literature there are only heterogeneous examples (Table 1), which give the idea of how confusing the situation is [3-6]. The purpose of this paper is to summarize the state of the art of intraoperative fluid therapy and fluid approach today.

**Table 1:** Heterogeneous protocol

	**Liberal**	**Restricted**
*Holte et al.*[3]	30 mL/Kg/h	10 mL/Kg/h
*Holte et al.*[4]	18 mL/Kg/h RL + 7 mL/Kg/h HES 130/0.4	5-7 mL/Kg/h RL + 7 mL/Kg/h HES 130/0.4
*Abraham-Nordling M. et al.*[5]	5 mL/Kg/h RL + 2 mL Gluc 2.5%	2 mL/Kg/h Gluc 2.5%
*Lobo S. et al.*[6]	12 mL/Kg/h RL	5 mL/Kg/h RL

## Discussion

### *Pathophysiological aspect*

The consequences of inappropriately high fluid administration may be significant, relating to liberation of atrial natriuretic peptide and an iatrogenic glycocalyx/vascular endothelial junction dysfunction, which leads to fluid shifts into the extravascular space [7]. This pathologic shift is caused by a dysfunction of the vascular barrier basically because of 3 reasons: surgical manipulation, reperfusion injury and iatrogenic hypervolemia (regardless of kind of fluids administered, crystalloids or colloids). Chappell et al. described this type of fluid shifting toward the interstitial space as follow [8]:

– Type 1 disorder: represents an almost colloid-free shift of fluids and electrolytes out of the vasculature, even if the vascular barrier is intact (i.e. if large amounts of isotonic crystalloids are infused);

– Type 2 disorder: the fluids shift contains proteins close to the plasma concentration, crossing a functionally altered vascular barrier; this occurs inconstantly and is related to the type, extent, and duration of surgery and also the type of fluid used (crystalloids versus colloids).

### Type of fluids and patients population

Normal saline solution (the most used crystalloid) has been used for over 50 years as an intraoperative, resuscitation and maintenance fluid; however its excessive use can lead to hyperchloremic acidosis and type I disorder. There is currently a debate regarding the morbidity associated with this condition, although its incidence is considered to be very low [9-11]. The British Consensus Guidelines on Intravenous Fluid Therapy for Adult Surgical Patients recommend the use of balanced crystalloids rather then just saline solution to avoid hypercloremic acidosis [12].

The dispute in literature about fluids, as reported in the 6S, CRYSTMAS and CHEST studies, clearly highlights the problem of the use of the right fluid in the right setting (intensive care) and for the right patient (septic patient and hemodynamic unstable intensive care unit (ICU) patients) [13-15]. These three large randomized clinical trials evaluated outcome and adverse effects of fluid resuscitation in septic patients comparing hydroxyethyl starches (HES) versus normal saline or Ringer acetate’s solution: the result is that no difference in mortality has been observed between the different types of fluids (except in 6S study) [13]. In terms of kidney injury, CHEST and 6S trials revealed that in HES group there is a greater risk of developing renal dysfunction; 6S trial is the only one study that evaluate HES 130/0.42 (tetraspan); but the CRYSTMAS trial did not reveal any difference in terms of adverse events in both fluid groups [15]. The CRISTAL study has found instead that the use of colloids, compared to crystalloids, has resulted in a reduction of mortality at 90 days (not at 28 days) [16].

Finally, the protocols of these studies need to be discussed [17].

But the question is: are there any differences between a “relatively” healthy patient, like who is undergoing elective surgery, and a critically ill patient? In the first case the intact tight glycocalyx/vascular endothelial junction provide a proper retention of colloids, while in the second scenario the endotoxic shock or the generalized inflammatory response can lead to the disruption of the vascular barrier integrity, causing altered distribution of large molecules [18-20]. Many anesthetists use colloids solutions, mostly (HES), for blood volume expansion [21]; among these the so-called tetrastarch (a third generation hydroxyethyl starch 130/0.4) seems to have a better safety profile [22,23] and there is no added risk to develop acute kidney injury in patients who receive HES during operation time [24]. In a recent review, Van Der Linden et al. [25] analyzed the safety of modern starches used during surgery: the authors concluded by arguing that the use of starches during surgery do not induce adverse renal effects, do not increase blood loss, do not require more allogenic erythrocyte transfusions and do not increase mortality [25]. But once again colloids need to be administered at the right time for the right patient; its prophylactic use to anticipate acute bleeding or to extend intravascular blood volume in a primary normovolemic patient should no longer be considered state-of-the-art. Colloids should be used to replace blood loss.

### Type and duration of surgery

**Figure 1:**
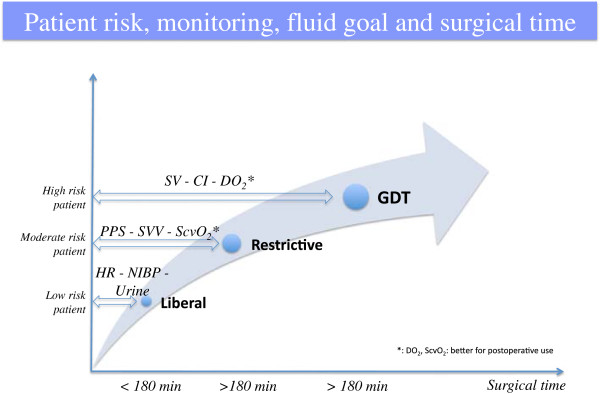
**Patient monitoring.** Hemodynamic monitoring need to be considered on the basis of patient risk, surgical type and time.

**Figure 2:**
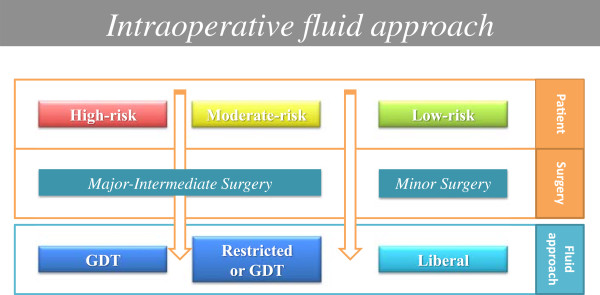
**Perioperative fluid therapy.** Intraoperative fluid therapy must take into account patient risk and type of surgery.

The type 2 disorder, as described by Chappell, opens the discussion about the type and duration of surgery (Figure 1) [8]. Now a differentiation between major and minor operations as well as abdominal versus non-abdominal surgery seems to be necessary. In a high risk surgical patient [19] undergoing an intermediate to major risk surgery some evidence based medicine support the application of a goal directed therapy (GDT), in which fluid administration is targeted on hemodynamic parameters (i.e. stroke volume) with the aim to maximize the oxygen delivery and then avoiding oxygen debt (Figures 1 and 2) [26,27]. This approach should be the best thing to do, but there are limitations that remain a major obstacle: the invasiveness of pulmonary artery catheter and transplumonary dilution technique and the poor accuracy and precision of the non-invasive devices (Vigileo - Edwards Lifesciences, Irvine, CA, USA; LiDCO - LiDCO Ltd, Cambridge, UK; ecc.) [28,29]. On the other hand, in moderate to high risk patient who is scheduled to undergo major surgery expected to last > 180 minute, a GTD approach with the optimization of hemodynamic parameters could reduce complications [8,30].

Historically, thoracic surgery has been the first married with the restricted fluid approach, but recently the emergence of new data shows that the risk of renal insufficiency after lung resection surgery is about 6-24%. So, in this setting, is necessary to specify two major branch: in patients undergoing pneumonectomy the restrictive approach seems to be up-to-date but for lesser resection a GTD approach should be considered [31].

In clinical practice many studies investigated if a restrictive approach can improve surgical outcome, especially in major abdominal surgery. Lobo et al. demonstrated an improved gastrointestinal function after elective colonic resection; they also demonstrated a reduced length of hospital stay (LOS) from 9 to 6 days in the restrictive group [32]. Brandstrup demonstrated that in colo-rectal surgery a restricted management reduced post-operative complications and death [33]. Some studies in vascular patients undergoing major abdominal aortic surgery failed to identify specific superior fluid regimens. However, in this type of surgery, there is a convincing and independent association between fluid accumulation and duration of ventilation, duration of ICU stay, ICU and in-Hospital mortality and development of complications as well, such as acute respiratory distress syndrome and acute kidney injury [34]. In that context, individualized fluid administration guided by flow parameters (GDT) seems to be the best (GDT works best if it is obtained by flow algorithms) [35,36]. However we must keep in mind that the hemodynamic management with the GDT tends to lead to “maximization” and not “optimization”, which are two different concepts; and that routine cardiovascular monitoring such as blood pressure, heart rate and urine output are not reliable predictors of intravascular fluid status and thus not a rational guide to perioperative fluid therapy.

Finally, several studies seem to suggest that in low-risk patients undergoing minor to intermediate risk surgery, liberal strategy (non restrictive) may be preferable. It reduces some postoperative complications such as nausea, vomiting, drowsiness, dizziness and length of stay [2,37-39]. In patients undergoing minor surgery, mostly in the ambulatory setting, liberal fluid administration may improve early recovery and symptoms of dehydratation (dizziness, nausea and thirst) [40,41].

### The third fluid administration strategy in the perioperative setting: goal-directed therapy

The GDT is an approach focused on the use of cardiac output (CO) and related parameters as end-points for fluids and drugs to optimize tissue perfusion and oxygenation by maximizing oxygen delivery (DO2). This technique was originally applied in surgical patients to achieve normal or supranormal values of CO and DO2 to prevent the oxygen debt caused by the perioperative increase in oxygen consumption [42]. Several clinical trials, metanalysis and reviews demonstrated its effectiveness, thus leading numerous societies to publish official guidelines that recommend its use in high-risk surgical patients [43-46].

Many tools can be used for GDT approach but the esophageal doppler (ED) monitoring (CardioQ-ODM, Deltex Medical, Chichester, West Sussex, UK) is the most supported by the literature. There are eight randomized controlled trials investigating ED-guided fluid administration: two in cardiac [47,48], two in orthopedic [49,50] and four in abdominal surgery [51-54]. These studies involve the use of a protocol designed to optimize the management of fluids through parameters such as stroke volume (SV) and flow time corrected (FTc), both during surgery and for the first 6–8 postoperative hours. The results show a reduced postoperative length of stay and a decrease in postoperative morbidity. As a result of these trials, ED-guided fluid administration has been recommended as routine for colorectal surgeries in the UK and endorsed by Medicare and Medicaid Service [55,56].

Few studies investigated the use of other advanced hemodynamic monitoring tools (such as pulmonary and trans-pulmonary thermodilution) for intraoperative GDT, probably because of their invasiveness resulting in limited use in the operating room. Sandham et al. studied the use of the pulmonary artery catheter (PAC) on patients classified ASA (American Society of Anesthesiology) 3 and 4 but failed to demonstrate benefits regarding length of hospital stay and mortality [57].

In recent years the concept that the oxygen debt leads to an increased incidence of postoperative complications (infections, organ failure, etc.) prompted many manufacturers to develop alternative less-invasive hemodynamic devices. The most widespread of these use the uncalibrated pulse-contour analysis, a technique developed from the original algorithm described by Wesseling [58], which calculates CO by assessing the area under the arterial curve (i.e. Vigileo, Edwards Lifescience, Irvine, CA, USA; Pulsioflex, Pulsion Medical Germany). These instruments continually estimate CO and derived parameters, are easy to use and are less invasive. However, they are also less accurate and precise than those calibrated. LiDCO rapid (LiDCO, London, UK), is different from the others as it uses an algorithm based on the pulse power analysis [59].

Clinical trials that compared the reliability of these uncalibrated pulse contour analysis systems show conflicting results [60-63]. In a multicenter study, Salzwedel et al. have shown that performing hemodynamic optimization using ProAQT/PulsioFlex (PULSION Medical Systems SE, Munich, Germany) led to a decrease in postoperative complications in patients undergoing major abdominal surgery [64]. On the other hand Pearse and the OPTIMISE Study Group were recently unable to demonstrate any advantage in using GTD in high-risk patients undergoing major gastrointestinal surgery, compared to standard care [65]. However the inclusion of those data in an up-to-date meta-analysis (including studies from 1988 to 2013) indicates that the intervention was associated with a reduction in overall complication rates. This may be attributed to the fact that the same author, in a previous study on GDT guided by the calibrated LiDCO (LiDCO plus system; LiDCO Ltd., Cambridge, UK), showed improved outcomes after major surgery [66]. In the last study Pearse and coll. [66] used LiDCO rapid, which is an uncalibrated thermodilution technique to measure CO, quite different from LiDCO plus (that uses calibrated evaluation). However that paper is the only one that enrolled and studied the highest number of patients to evaluate outcome with or without GTD (65).

Hemodynamic devices are not interchangeable and ED-guided fluid therapy has been shown to be superior to a liberal fluid strategy but not to a restrictive approach [67]. Anyway doubts have been raised on the quality of the studies supporting the ED in a recent letter [68], with the conclusion that further studies are necessary to confirm or refute this evidence.

We have to mention also the non-invasive technologies: the Nexfin (BMEye, Edwards Life Sciences, Amsterdam, The Netherlands) and the Masimo (Masimo Corporation, Irvine, CA, USA). The Nexfin device uses a finger pneumatic cuff to evaluate a continuous non-invasive arterial pressure curve, through which it estimates the CO by pulse contour analysis. The Masimo provides a non-invasive estimation of the plethysmography variability index (PVI) as a consequence of variation in peripheral pulse oximetry, which has been shown to be related to pulse pressure variation (PPV). PVI is a good predictor of fluid responsiveness and has proven to be a reliable indicator in cardiac and colorectal surgery, but only in the context of a hemodynamically stable patient [69,70].

What is still missing in the literature is to determine whether different instruments play different roles and if different populations of patients (ASA II-III versus IV) can benefit from these techniques. Finally, what is really important for GTD is its fundamental (patho) physiological concept: pay the “fluid debt” but also pay it “on time”, within the intraoperative and first 6–8 postoperative hours.

A recent survey [71] shows that the use of GDT differs according to the medical and anesthesiological culture promoted in different settings: it is more widespread in the UK compared to the Australian/New Zealand. The majority of the respondents were involved in major abdominal and orthopedic surgery and used GDT in patients with significant comorbidities. The most significant barriers that hamper the adoption of the GDT were either a lack of availability of monitoring tools or a lack of experience with these instruments [72].

There is a long-standing debate on this manipulation of oxygen delivery in high-risk surgical patients: a therapeutic option may be not to stop at the achievement of the normalization of hemodynamic values, but to go further and aim for a “supranormal oxygen delivery”, described as the therapeutic goal of a DO_2_I > 600 ml/min/m2. This trend began in the ‘70s and ’80s with the “milestone” of Shoemaker [73], showing that the induction of a hyperdynamic cardiovascular status was protective reducing the postoperative morbidity and mortality. However there is no lack of criticism of this approach, as it has been revised only by single-center studies with unintentional bias due to lack of blinding and different inclusion criteria and definitions. Concerning the complications, the most feared are those related to invasive monitoring and cardiac complications associated with fluid challenges and inotropes administration. Other studies failed to show any survival benefit attributable to supranormal GDT [74].

A recent metanalysis has shown that the benefit of GDT is most pronounced in patients receiving fluid and inotrope therapy to achieve a supranormal oxygen delivery target, with the use of minimally invasive cardiac monitors [75].

Perioperative optimization can be guided by lactates levels too: the development of an imbalance between oxygen delivery and oxygen consumption, define as oxygen debt, lead to lactates production above normal levels. Some studies showed that perioperative optimization targeted at lowering lactates levels can reduce post-operative complications [76].

## Summary

**Figure 3:**
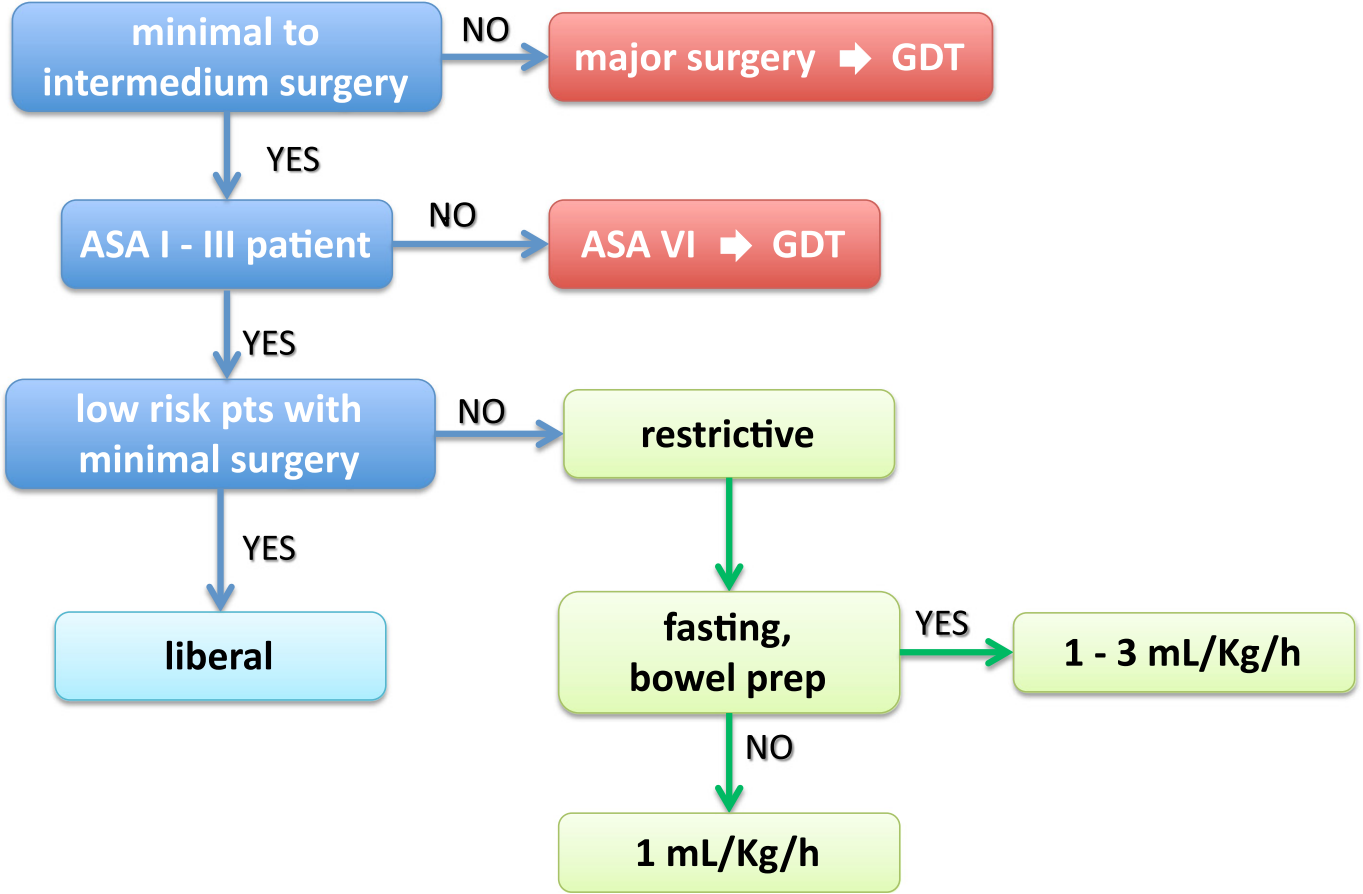
**Fluid flow-chart.** The steps reported represent a rational approach to fluid management in ASA I-III patients.

Many studies demonstrate that an intraoperative restrictive fluid approach in major surgery improves outcome, reduces length of hospital stay, reduces anastomotic leakage and surgical site infection [5,33-41,77]. In a systematic review of 80 randomized clinical trials, Holte and Kehlet [78] recommended to avoid fluid overload in major surgical procedures. The approach to the intraoperative fluid management is complex and it should be based on human physiology and the current literature. The steps reported in Figure 3 represent a rational approach to fluid management in ASA I-III patients. The literature evidence available up to date suggests that GDT should be superior to the liberal fluid strategy; until there will be evidence that the GTD is superior or not to the restrictive fluid therapy the following approach became rational:

• During intraoperative period the anesthetist should give as many fluids as required by that single patient. Either hypovolemia, that causes organ hypoperfusion, and hypervolemia, which increases postoperative complications, should both be avoided;

• In all patients **ASA I-III** undergoing surgery, excluding cardiac surgery or transplantation surgery, 1 mL/kg/h of crystalloid solution should be given (if the patient did not observe the overnight fasting or did not make bowel preparation);

• In fasted patient or those who underwent bowel preparation, 1–3 mL/kg/h should be given;

• In all ASA IV patients and/or in those who are undergoing high risk surgery a GDT is strongly suggested;

• Blood loss has to be replaced only by colloids until the hemoglobin does not reach a value of 7 g/dL (if the patient does not have cardiovascular or respiratory coexisting diseases);

• In case of diuresis monitoring, its total amount will be replaced by balanced crystalloid solutions (unless contraindicated);

• In case of hypotension soon after general anesthesia induction or during intraoperative period, it is necessary to check anesthesia level and then use vasopressors before administering fluids;

• In the postoperative period it is mandatory to restore oral hydratation and feeding as soon as possible (unless contraindicated).

## Abbreviations

ASA: American society of anesthesiology physical status classification system; CO: Cardiac output; DO2: Oxygen delivery; ED: Esophageal doppler; FTc: Flow time corrected; GDT: Goal directed therapy; HES: Hydroxyethyl starch; ICU: Intensive care unit; LOS: Length of stay; PAC: Pulmonary artery catheter; PPV: Pulse pressure variation; PVI: Pleth variability index; SV: Stroke volume.

## Competing interests

The authors declare that they have no competing interests.

## Authors’ contributions

GDR, GT and LV planned the paper and drafted the manuscript and created the flow-charts of the paper and helped to find the bibliography. LP, FB, CD helped to find the bibliography and drafted the manuscript. All authors read and approved the final manuscript.

## Pre-publication history

The pre-publication history for this paper can be accessed here:

http://www.biomedcentral.com/1471-2253/14/62/prepub
